# Thermodynamic origin of instability in hybrid halide perovskites

**DOI:** 10.1038/srep37654

**Published:** 2016-11-24

**Authors:** E. Tenuta, C. Zheng, O. Rubel

**Affiliations:** 1 Department of Materials Science and Engineering, McMaster University, 1280 Main Street West, Hamilton, Ontario L8S 4L8, Canada

## Abstract

Degradation of hybrid halide perovskites under the influence of environmental factors impairs future prospects of using these materials as absorbers in solar cells. First principle calculations can be used as a guideline in search of new materials, provided we can rely on their predictive capabilities. We show that the instability of perovskites can be captured using *ab initio* total energy calculations for reactants and products augmented with additional thermodynamic data to account for finite temperature effects. Calculations suggest that the instability of CH_3_NH_3_PbI_3_ in moist environment is linked to the aqueous solubility of the CH_3_NH_3_I salt, thus making other perovskite materials with soluble decomposition products prone to degradation. Properties of NH_3_OHPbI_3_, NH_3_NH_2_PbI_3_, PH_4_PbI_3_, SbH_4_PbI_3_, CsPbBr_3_, and a new hypothetical SF_3_PbI_3_ perovskite are studied in the search for alternative solar cell absorber materials with enhanced chemical stability.

The search for cost-effective solar cell absorber materials that can compete with the performance of crystalline silicon and thin-film (GaAs, CdTe and Cu(In, Ga)Se_2_) solar cells remains the priority for renewable energy material research. A recently emerged class of hybrid halide perovskite materials holds a promise to lead the way towards low-cost photovoltaic devices as they combine an energy conversion efficiency of nearly 20% with a low-temperature solution processing technology^1^,^2^,^3^,^4^. The structure of hybrid perovskites 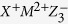 is formed by a combination of various organic cations *X*^+^ = (CH_3_NH_3_, NH_4_, CH_5_N_2_), metallic cations *M*^2+^ = (Pb, Sn), and halide anions *Z*^−^ = (I, Cl, Br) with CH_3_NH_3_PbI_3_ being a prominent example. Perovskite materials possess a unique combination of characteristics that make them useful in photovoltaic applications including a favourable band gap of about 1.5–1.6 eV, efficient optical adsorption, long lifetime of optical excitations, and high level of mobility for charge carries of both polarities^5^,^6^,^7^,^8^.

A major weakness of perovskite solar cells is degradation of the power conversion efficiency in moist environment^9^,^10^. This degradation can be observed in CH_3_NH_3_PbI_3_ cells through the colour changing from black to yellow accompanied by a noted decreases in absorption and deterioration of the overall cell performance over time^11^,^12^. The absorbance at 410 nm has been reported to decrease by 50% after 4 hours of exposure to environment with the relative humidity of 98%^12^. The same study linearly extrapolated from the previous result concluded that the identical degradation would take approximately one year at the relative humidity of 20%^12^.

Frost *et al*.^13^ proposed an acid-base chemistry mechanism to explain the role of water in the degradation process. In this process the decomposition is driven by protic properties of the [CH_3_NH_3_]^+^ ion, thus suggesting that aprotic hybrid perovskites (e.g., (CH_3_)_4_NPbI_3_) could potentially be more stable^13^. However, this strategy was not confirmed experimentally to the best of our knowledge.

Density functional theory (DFT) simulations of the perovskite-water interface^14^,^15^ provided further insight to kinetics of the degradation mechanism at the atomic scale. Mosconi *et al*.^14^ observed dissolution of iodine cage and subsequent release of methylammonium ions as well as incorporation of water molecules in the perovskite structure at the interface. The simulation results reported by Zhang and Sit^15^ indicate deprotonation of methylammonium as an initial step in dissolution of the perovskite. Furthermore, first principle calculations^16^ performed without taking into account solvent effects suggest that hybrid halide perovskites may be intrinsically unstable. This conclusion is based on a nearly zero enthalpy of reaction associated with decomposition of the perovskite structure, which is evaluated based on the total energy of reactant and products. Frost *et al*.^13^ attributed the intrinsic instability of halide perovskites 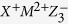 to its relatively low Madelung lattice energy as compared to oxide perovskites that belong to 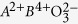 family. This result implies that the environmental factors (such as moisture, UV radiation, and elevated temperatures) may only accelerate the decomposition process. Therefore, the effectiveness of encapsulation as a strategy to prevent moisture damage may not guarantee a long-term stability of perovskite solar cells as evidenced by Han *et al*.^17^.

Here we utilize DFT to explore stability of perovskite structures from thermodynamic perspective. We will show that the finite temperature effects, that are omitted in former calculation of stability of CH_3_NH_3_PbI_3_^18^,^19^, can play a decisive role when the decomposition reaction takes place in the presence of a solvent and yields a water-soluble product. We will also examine properties of several alternatives, NH_3_OHPbI_3_, NH_3_NH_2_PbI_3_, PH_4_PbI_3_, SbH_4_PbI_3_, and CsPbBr_3_, as well as a hypothetical material, SF_3_PbI_3_, in a search for perovskite compounds with an enhanced chemical stability for photovoltaic applications.

## Results and Discussion

### Chemical stability of CH_3_NH_3_PbI_3_

We begin by examining the chemical stability of CH_3_NH_3_PbI_3_ against decomposition. The structural changes in the course of degradation involve disappearance of X-ray diffraction peaks that are characteristic for CH_3_NH_3_PbI_3_ and appearance of PbI_2_ peaks^20^, which suggests the following reaction (phases)


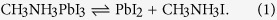


The standard approach for predicting the direction of a chemical reaction involves evaluation of the change in the Gibbs free energy between reactants and products (see ref. 21, Chap. 7), which can be split into two terms for convenience


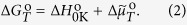


Here 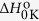 is the standard reaction enthalpy change at zero temperature, and 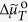 captures finite temperature effects on the chemical potentials of species involved.

The enthalpy change at zero temperature can be readily evaluated based on the DFT total energy calculations


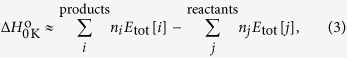


where *n* and *E*_tot_ are the number and the total energy of the chemical species involved in the reaction. It should be noted that the bare DFT total energies in Eq. (3) do not fully capture the standard enthalpy changes at zero temperature as they do not include a zero-point vibrational energy and, less importantly, the standard pressure effects on *E*_tot_. Therefore, the relation (3) is approximate. In the case of CH_3_NH_3_PbI_3_, the decomposition reaction yields two products: PbI_2_ and CH_3_NH_3_I. The corresponding lattice parameters and total energies of the reactant and products are listed in Table 1. The values yield the dissociation reaction enthalpy of 
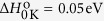
 per formula unit (f.u.) for CH_3_NH_3_PbI_3_ evaluated using Eq. (3). The result is within the range of theoretical values reported in the literature, including 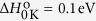
22 and −0.06 eV^16^. The corresponding experimental value is 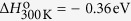
23, which is far too low to explain formability of this perovskite and needs further experimental verifications. The theoretical value of 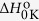 contrasts sharply with the formation enthalpy of major solar cell compound materials, such as GaAs and CdTe, which is of the order of 0.8–1 eV/f.u.^24^,^25^.

The poor chemical stability of CH_3_NH_3_PbI_3_ is often attributed to the nearly vanishing value of Δ*H*^16^,^18^. In fact this only implies that the decomposition does not involve a heat exchange with environment. It is the finite temperature contribution to the chemical potential difference between reactants and products


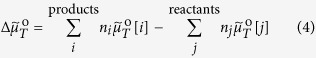


that remains overlooked in previous stability analysis^18^,^19^,^23^. Here 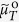 represents the final temperature correction to the chemical potential of spieces


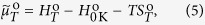


which is not captured in a bare DFT total energy. Although the extension of DFT calculations to finite temperatures is possible^26^, it is computationally intensive. Therefore, NIST-JANAF thermochemical tables as well as other experimental resources were used to evaluate the final temperature correction using Eq. (5) (see Table 1 and references therein).

The final temperature correction to the Gibbs free energy of CH_3_NH_3_PbI_3_ decomposition reaction


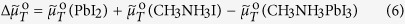


amounts to 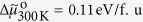. The resultant Gibbs free energy difference in Eq. (2) is positive 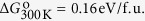 indicating that the final temperature effects tend to stabilize the perovskite structure against spontaneous decomposition under standard conditions for temperature and pressure. However, the result should be taken with caution, since the uncertainty in reaction energies obtained with Perdew, Burke, and Ernzerhof (PBE)^27^ exchange-correlation functional is of the order of ±0.03 eV/atom^28^.

The thermodynamic characteristics of CH_3_NH_3_PbI_3_ perovskites indicate that its chemical stability is fragile, and the balance can be easily shifted if the environment changes. A possible scenario that will be discussed here involves presence of a solvent. Unlike PbI_2_ that has a limited solubility in water, the methylammonium iodide is highly soluble in water, which should be taken into account when calculating its chemical potential (see ref. 29, Chap. 8).

The actual chemical potential of an electrolyte


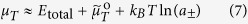


can be significantly different from its value in the standard state depending on the activity coefficient *a*_±_ of the solute^30^. The mean ionic activity *a*_±_ of CH_3_NH_3_I solution


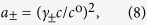


is determined by its molar concentration *c* relative to the concentration in standard state *c*^o^ = 1 M and the mean ionic activity coefficient *γ*_±_, which account for non-ideality of the solution. In a dilute solution limit, 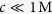 and *γ*_±_ ~ 1, the chemical potential of aqueous CH_3_NH_3_I drops much below its value in the solid phase


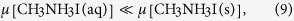


which shifts the balance in Eq. (1) to the right. Accordingly, the aqueous solubility of methylammonium iodide drives the dissociation of CH_3_NH_3_PbI_3_ as previously suggested by Niu *et al*.^20^. The decomposition proceeds as long as the following condition is fulfilled


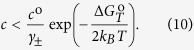


Using the value of 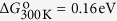 and assuming *γ*_±_ ~ 1, it is possible to estimate the saturation concentration *c*_s_ of CH_3_NH_3_I dissolved in water at which further decomposition of CH_3_NH_3_PbI_3_ is suppressed. Equation (10) yields *c*_s_ ~ 50 mM (or ~8 g/L). Given the fact that the thickness of the absorbing material in perovskite solar cells is only 0.5 *μ*m^31^, even a droplet of water is sufficient to destroy a device with the area of a several square centimeters.

### Alternative absorber materials

The stability of hybrid lead halide perovskites can be improved by substituting iodine with more electronegative elements (bromine or chlorine)^23^. However, the associated increase of the band gap that exceeds 2 eV^22^ limits the accessible power conversion efficiency when aiming for solar cell absorber materials. Therefore, we focus on perovskite structures of the family *X* PbI_3_ and explore several alternatives for the cation *X* = [NH_3_OH]^+^, [NH_3_NH_2_]^+^, [PH_4_]^+^, [SbH_4_]^+^, and [SF_3_]^+^. A solid solution of hydroxylammonium and hydrazinium ions were recently used in hybrid perovskite structures^32^. Phosphonium and particularly stibonium ions were theoretically predicted to produce more efficient photovoltaic materials when substituted for methylammonium in lead iodide-based perovskites due to the reduced band gap and improved effective mass^33^. Sulphur trifluoride represents an aprotic cation that can be beneficial in the context of resistance to degradation according to the mechanism discussed by Frost *et al*.^13^. Similar ionic volumes of [SF_3_^+^] (0.053 nm^3^) and [CH_3_NH_3_]^+^ (0.051 nm^3^) indicate proximity in size of both ions^34^.

Unit cell volumes and band gaps of the corresponding perovskite structures are listed in Table 2. The band gaps were calculated without taking into account relativistic effects. This approach allows to minimize the error by taking advantage of an error cancelation between the band gap reduction due to spin-orbit coupling and its opening introduced by a port-DFT correction^35^. As a result, values of the band gap are only slightly overestimated (approximately 0.1 eV). The results indicate that small changes in the volume (less than 5%) lead to a sizeable change in the band gap. In contrast to group IV, III-V and II-VI semiconductors, the band gap in perovskite structures *increases* when the unit cell expands as Dittrich *et al*.^8^ noticed. Out data clearly follow this trend with SbH_4_PbI_3_ being a favourite candidate for single-junction solar cells due to proximity of its band gap to the ideal value of 1.4 eV, which corresponds to the maximum efficiency in the Shockley-Queisser limit^36^.

The chemical stability of perovskite structures in Table 2 was initially assessed by computing the decomposition reaction enthalpy 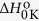. In this calculation, we shall assume that all structures decompose following the pathway similar to Eq. (1) with the exception of SF_3_PbI_3_. Since there are no reports in the literature for SF_3_I salt, the following decomposition route is considered


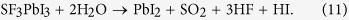


This decomposition route also involves water, but in a different capacity from the degradation of CH_3_NH_3_PbI_3_. Here water directly reacts with the perovskite.

Results for the decomposition reaction enthalpy calculated using Eq. (3) are given in Table 2, where the compounds are sorted in the order of increasing 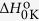 (higher values favour stability of perovskites). Among all hybrid perovskites listed in Table 2, SF_3_PbI_3_ shows the highest decomposition reaction enthalpy indicating that the reaction (11) is strongly *endothermic*. All other hybrid perovskites with the negative reaction enthalpy can be rendered as unstable, including SbH_4_PbI_3_ with the promising band gap value.

**Figure 1:**
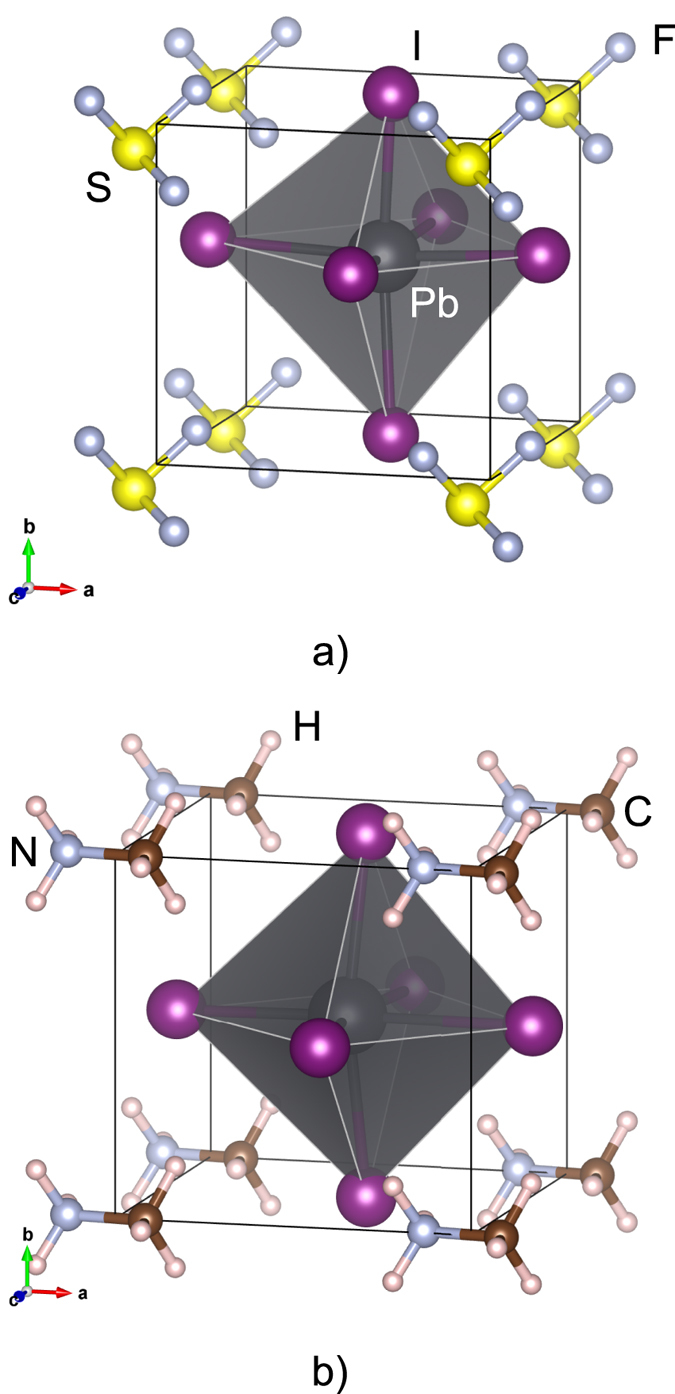
Structure of pseudocubic SF_3_PbI_3_ (**a**) and CH_3_NH_3_PbI_3_ (**b**).

**Figure 2:**
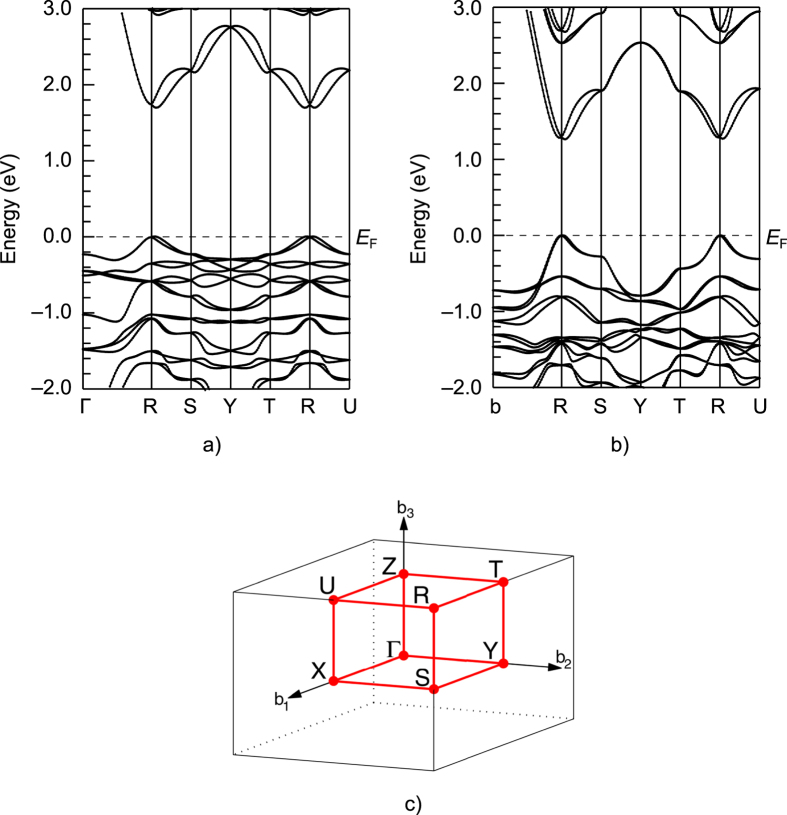
Band structure of pseudocubic SF_3_PbI_3_ (**a**) and CH_3_NH_3_PbI_3_ (**b**) calculated along the path between high-symmetry points in the Brillouin zone (**c**) taking into account spin-orbit coupling and non-local exchange correction. The origin of the energy scale is set at the Fermi energy *E*_F_. Labels of the high-symmetry point in the Brillouin zone correspond to an orthorhombic lattice^53^.

The pseudocubic structures of SF_3_PbI_3_ perovskite is shown in Fig. 1 alongside with the pseudocubic structure of CH_3_NH_3_PbI_3_. The band structure of both materials calculated taking into account spin-orbit coupling and a meta-GGA band gap correction are presented in Fig. 2. Both structures share qualitative similarities of the band dispersion. The band gap of SF_3_PbI_3_ is 0.4 eV higher than that for CH_3_NH_3_PbI_3_. It is tempting to conclude that the new compound SF_3_PbI_3_ is stable due to the positive value of the enthalpy. It should be emphasized, however, that calculations of stability based on the formation enthalpy alone can lead to spurious results. If we take into account the final temperature correction 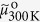 for the reactants and products in Table 1, we obtain the Gibbs free energy difference of 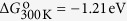. The negative value suggests that the reaction (11) can proceed spontaneously. This renders SF_3_PbI_3_ as being susceptible to reaction with moisture and warrants encapsulation as a protective provision against degradation.

Finally, it will be instructive to discuss the stability of an inorganic CsPbBr_3_ perovskite. Unlike CH_3_NH_3_PbI_3_, the decomposition reaction enthalpy of CsPbBr_3_ is high (Table 2). Assuming that both compounds have the same magnitude of the final temperature contribution 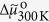 to the free energy, one would expect the free energy of CsPbBr_3_ to be approximately 0.5 eV/f.u. *lower* than that for decomposition products (PbBr_2_ and CsBr) indicating strong chemical stability of CsPbBr_3_ against spontaneous decomposition. However, it is found experimentally that the performance of CsPbBr_3_-based solar cells (not encapsulated) decays over time, although slower than CH_3_NH_3_PbI_3_-based devices^37^. This observation reveals susceptibility of both perovskite structures to the reaction with moisture, despite of the high reaction enthalpy of CsPbBr_3_ and lack of proton-donating groups. We believe that the aqueous solubility of CsBr has some significance for explaining this effect. The slow degradation rate of CsPbBr_3_ can be attributed to the greater value of 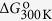, which translates into a much lower saturation concentration of CsBr *c*_s_ ~ 60 *μ*M as compared to *c*_s_ ~ 50 mM for CH_3_NH_3_I (see discussion in the preceding subsection).

## Conclusions

The performance of CH_3_NH_3_PbI_3_ perovskites solar cells deteriorates when exposed to environmental factors, such as moisture and sunlight. This remains the main barrier on the way to their commercialization. The ability to assess stability of solar cell absorber materials using first principle calculations is an important attribute for design of new materials. We showed that the instability of perovskites can be captured using DFT total energy calculations for reactants and products augmented with additional thermodynamic data to account for finite temperature effects. The finite temperature effects play a minor role stabilizing the perovskite structure when products of the decomposition reaction are solids. However, the finite temperature contribution to the Gibbs free energy of the degradation reaction becomes crucially important in the case of when products of the decomposition are aqueous solutions or gases.

Our calculations suggest that the CH_3_NH_3_PbI_3_ structure can be stable against spontaneous decomposition, provided it is isolated from environmental factors. The situation changes drastically in the presence of water. The aqueous solubility of the CH_3_NH_3_I salt lowers its chemical potential relative to the solid phase, especially in a dilute limit. This property favours decomposition of CH_3_NH_3_PbI_3_ in the moist environment. Therefore, a limited solubility of the decomposition reaction products is anticipated to improve structural stability. Generalizing this result to other perovskites, the aqueous solubility of HC(NH_2_)_2_I, SbH_4_I, and CsBr undermines stability of the corresponding perovskite structures.

In a search for alternative perovskites, NH_3_OHPbI_3_, NH_3_NH_2_PbI_3_, PH_4_PbI_3_, SbH_4_PbI_3_, CsPbBr_3_, and SF_3_PbI_3_ compounds were investigated. NH_3_OHPbI_3_, NH_3_NH_2_PbI_3_, PH_4_PbI_3_, and SbH_4_PbI_3_ were concluded unstable due to the low formation enthalpy. The CsPbBr_3_ structure is prone to degradation in moist conditions, in spite of the favourable formation enthalpy, due to solubility of CsBr. Straightforward comparison of DFT total energies of reactants and products provides an argument in favour of the chemical stability for the SF_3_PbI_3_ structure. The predicted value for the energy band gap of this new compound is approximately 2 eV. However, more detailed analysis that incorporates finite temperature effects renders the material unstable to decomposition in a moist environment thus signifying the importance of those effects for future analysis.

### Computational details

The first-principles electronic structure calculations were carried out using DFT^38^. Two implementations were employed. A projector augmented wave (PAW) method^39^,^40^ implemented in the ABINIT package^41^,^42^,^43^ was utilized for the structural optimization and calculations of the chemical stability. The band structure calculations were performed in the Wien2k package^44^ based on a full potential linear augmented plane wave method.

### Structure optimization

The CH_3_NH_3_PbI_3_ perovskite is known to exist in three different polymorphs^5^,^45^: orthorhombic, tetragonal, and cubic. A tetragonal *β*-phase is stable at room temperature and was used in these calculations. A pseudocubic structure was used to represent SF_3_PbI_3_, NH_3_OHPbI_3_, NH_3_NH_2_PbI_3_, PH_4_PbI_3_, and SbH_4_PbI_3_. An orthorhombic (Pnma) structure was chosen to represent *δ*-CsPbBr_3_. Optimization of lattice parameters was carried out in conjunction with relaxation of internal degrees of freedom for all structures studied here. The structure was considered optimized when the magnitude of Hellmann-Feynman forces acting on atoms dropped below 0.5 mHa/Bohr and components of the stress tensor were less than 1 *μ*Ha/Bohr^3^. The Brillouin zone was sampled using an unshifted mesh with the density one k-point per every 0.01 Bohr^−1^ length of each reciprocal lattice vector. The cutoff energy for a plane wave expansion was set at 15 Ha.

Standard structures of solid PbI_2_ (hexagonal, space group 164 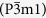
m1)), PbF_2_ (cubic, space group 225 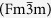) and PbBr_2_ (orthorhombic, space group 62 (Pbnm)) were used to represent possible reactants. The structure of CH_3_NH_3_I undergoes several phase transitions with increasing temperature^46^. A tetragonal *α*′-phase (space group 129 (P4/nmm)), which is stable at room temperature, resembles a rock-salt ionic structure^47^. The total energy of water was derived from its natural I_h_ solid structure (hexagonal, space group 194 (P6_3_/mmc) ref. 48). Structures of NH_3_OHI, NH_3_NH_2_I, PH_4_I, and SbH_4_I were derived using CsCl structure as a prototype. All structures were fully optimized as describe in the preceding paragraph (without constrains to the geometry).

Gaseous phases were modelled as an individual molecule surrounded by 30 Bohrs of vacuum. The internal degrees of freedom were relaxed. Only Γ-point was used in the Brillouin zone.

Perdew, Burke, and Ernzerhof^27^ version of the generalized gradient approximation was chosen for the exchange correlation functional due to its superior accuracy in predicting cohesive properties of solids and molecules.

Garrity, Bennett, Rabe, and Vanderbilt^49^ GBRV (v1.5) PAW pseudopotentials were employed for all elements. VESTA 3 package was used for visualization of atomic structure^50^. Structure files of all perovskite compounds and non-trivial salts are included in the supplementary information in a cif-format.

### Band structure

The band structure of pseudocubic SF_3_PbI_3_ and CH_3_NH_3_PbI_3_ were calculated with the Wien2k package^44^ using a full potential linear augmented plane wave method. The Brillouin zone was sampled using 6 × 6 × 6 Monkhorst and Pack^51^ mesh. The muffin-tin radii *R*_MT_ where set to 0.62, 1.16, 1.22, 1.38, 1.47, 2.2, and 2.2 Bohr for H, N, C, S, F, I, and Pb respectively. The cutoff energy of −6 Ry was used to separates valence and core electrons. The product 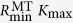, which determines the accuracy of a plane wave expansion of the wave function, was set at the values of 3.5 and 6 for CH_3_NH_3_PbI_3_ and SF_3_PbI_3_ compounds, respectively. The low 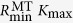 for CH_3_NH_3_PbI_3_ is due to a small size of the muffin-tin sphere around hydrogen atoms. Optimized lattice parameters and atomic positions from ABINIT calculations were used. The Tran-Blaha modified Becke-Johnson (TBmBJ) potential^52^ was applied in order to overcome shortcomings of DFT semilocal exchange correlation functions in predicting band gaps of insulators.

## Additional Information

**How to cite this article**: Tenuta, E. *et al*. Thermodynamic origin of instability in hybrid halide perovskites. *Sci. Rep.* **6**, 37654; doi: 10.1038/srep37654 (2016).

**Publisher’s note:** Springer Nature remains neutral with regard to jurisdictional claims in published maps and institutional affiliations.

## Supplementary Material

Supplementary Information

## Figures and Tables

**Table 1:** Equilibrium lattice parameters, electronic total energy *E*_tot_ per formula unit (f.u.), and change in the chemical potential 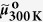 that accounts for the free energy of the compounds at the finite temperature and pressure not captured in DFT total energy.

Compound	Lattice parameter (Å)	*E*_tot_ (eV/f.u.)	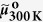 (eV/f.u.)
CH_3_NH_3_PbI_3_ (solid)	*a* = 8.92, *c*/*a* = 1.48^a^	−3146.596	−0.70^54^
PbI_2_ (solid)	*a* = 4.668, *c*/*a* = 1.63^b^	−2302.282	−0.34^55^
CH_3_NH_3_I (solid)	*a* = 5.146, *c*/*a* = 1.86^c^	−844.266	−0.25^56^
SF_3_PbI_3_ (solid)	*a* = 6.546	−4890.024	~−0.7^d^
H_2_O (solid)	*a* = 4.440, *c*/*a* = 1.63^e^	−471.805	−0.06^f^^ ^55,57
SO_2_ (gas)	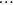	−1157.758	−0.66^55^
HF (gas)	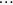	−679.866	−0.45^55^
HI (gas)	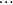	−333.125	−0.55^55^

^a^Experimental: *a* = 8.86 Å, *c*/*a* = 1.43^58^; other theoretical: *a* = 8.80 Å, *c*/*a* = 1.48^59^.

^b^Experimental: *a* = 4.557 Å and *c*/*a* = 1.53^60^.

^c^Experimental: *a* = 5.11 Å and *c*/*a* = 1.75^61^

^d^The value identical to CH_3_NH_3_PbI_3_ is used as an aproximation.

^e^Experimental: *a* = 4.5181 Å and *c*/*a* = 1.63^62^.

^f^The value includes contributions from solid and liquid phases that correspond to −0.05 and −0.01 eV, respectively.

**Table 2:** Dissociation reaction enthalpy 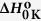 of perovskite structures presented together with volume of the unit cell *V*_0_ and the band gap energy 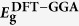 calculated self-consistently without taking into account the spin-orbit coupling.

Compound	*V*_0_ (Å^3^/f.u.)	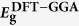 (eV)	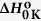 (eV/f.u.)^a^
NH_3_OHPbI_3_	270	1.89	−0.25
NH_3_NH_2_PbI_3_	274	1.80	−0.22
PH_4_PbI_3_	268	1.60	−0.18
SbH_4_PbI_3_	265	1.53	−0.11
*β*-CH_3_NH_3_PbI_3_	263	1.67	+0.05
*δ*-CsPbBr_3_	208	2.10	+0.37^b^
SF_3_PbI_3_	280	2.05	+0.87

The generalized reaction for chemical decomposition is given by Eq. (1), except for SF_3_PbI_3_ that decomposes following the pathway in Eq. (11).

^a^The positive value favours formation of the perovskite structure.

^b^0.21 eV/f.u. is an alternative DFT result reported by Zhang *et al*.^16^.
